# Spatiotemporal postural control deficits are present in those with chronic ankle instability

**DOI:** 10.1186/1471-2474-9-76

**Published:** 2008-06-02

**Authors:** Patrick O McKeon, Jay Hertel

**Affiliations:** 1 Department of Rehabilitation Sciences, University of Kentucky, Lexington, Kentucky, USA; 2 Department of Human Services, University of Virginia, Charlottesville, Virginia, USA; 3 Division of Athletic Training, University of Kentucky, College of Health Sciences, Wethington Building, Room 206C, 900 South Limestone, Lexington, KY 40536-0200, USA

## Abstract

**Background:**

Postural control deficits have been purported to be a potential contributing factor in chronic ankle instability (CAI). Summary forceplate measures such as center of pressure velocity and area have not consistently detected postural control deficits associated with CAI. A novel measurement technique derived from the dynamical systems theory of motor control known as Time-to-boundary (TTB) has shown promise in detecting deficits in postural control related to chronic ankle instability (CAI). In a previous study, TTB deficits were detected in a sample of females with CAI. The purpose of this study was to examine postural control in sample of males and females with and without CAI using TTB measures.

**Methods:**

This case-control study was performed in a research laboratory. Thirty-two subjects (18 males, 14 females) with self-reported CAI were recruited and matched to healthy controls. All subjects performed three, ten-second trials of single-limb stance on a forceplate with eyes open and eyes closed. Main outcome measures included the TTB absolute minimum (s), mean of TTB minima (s), and standard deviation of TTB minima (s) in the anteroposterior and mediolateral directions. A series of group by gender analyses of variance were conducted to evaluate the differences in postural control for all TTB variables separately with eyes open and eyes closed.

**Results:**

There were no significant group by gender interactions or gender main effects for any of the measures. There, however, significant group main effects for 4 of the 6 measures with eyes closed as the CAI group demonstrated significant deficits in comparison to the control group. There were no significant differences between groups in any of the TTB measures with eyes open.

**Conclusion:**

TTB deficits were present in the CAI group compared to the control group. These deficits were detected with concurrent removal of visual input. CAI may place significantly greater constraints on the sensorimotor system during single limb stance, resulting in a reorganization of postural control strategies. These deficits may be indicative of a diminished ability to respond effectively to changes in postural control demands in those with CAI.

## Background

Ankle sprains are among the most common injuries in the physically active population.[1] The most common predisposing factor to suffering an ankle sprain is a previous history of ankle sprain. [2-4] The subjective feeling of the ankle "giving way" after an initial ankle sprain and repetitive bouts of instability resulting in numerous ankle sprains has been termed chronic ankle instability (CAI).[5] CAI has been linked to many different causative factors including deficits in postural control.

Postural control deficits in single limb stance associated with CAI utilizing instrumented forceplate measurements have been reported in the literature. [6-9] However, there has not been consistency in the evidence as to whether these deficits can be captured with the use of these measures.[10] Traditionally, measures of center of pressure (COP) excursions which measure the temporal or spatial aspects of COP excursions such as velocity and range have been used to assess the corrective actions of the postural control system in order to maintain equilibrium in single limb stance. Investigators[6-9,11,12] using these traditional COP measures have not consistently detected significant differences in comparisons between CAI and control groups. A criticism of the traditional measures is that they may lack the sensitivity to detect postural control differences associated with CAI.[13]

A novel approach to assessing postural control differences related to CAI is quantification of time-to-boundary (TTB) measures, a spatiotemporal analysis of COP.[14] Rather than using summary measures of the entire COP data set, TTB utilizes the relationships of individual COP data points with the boundaries of support.[15] TTB takes into account both the velocity of COP excursion and the position on the foot where the excursion occurs. TTB represents the amount of time it would take the COP excursion to reach the boundary of support should the direction and velocity of the COP remain unaltered. A lower TTB value is indicative of postural instability as the individual has less time to respond due to high COP velocity and/or position close to the boundary of stability. Analyzing the spatiotemporal relationship of the COP to the boundary of stability has been shown to detect different aspects of postural control than traditional COP measures.[15] Changes in COP position and velocity in TTB analysis are relevant to their relationship to the boundary of the base of support. [13-17] A high velocity COP excursion when the COP is in the middle of the foot is less precarious than a high velocity excursion occurring close to the boundary of the foot.[13,15]

In a comparison of 15 females with CAI and 9 females without CAI, Hertel and Olmsted-Kramer demonstrated that the magnitude and variability of TTB measures in single limb stance were lower in the CAI group. Females with CAI had significantly less time to make a postural correction and approached the boundaries of support in a less variable manner than healthy females. These substantial decreases in TTB magnitude and variability were found in the absence of significant differences in traditional COP measures. It was hypothesized that this reduction in variability of TTB measures was related to a diminished ability to respond effectively to changes in postural control demands and indicated a more constrained sensorimotor system.[13] A limitation of this study was a relatively small and very homogeneous sample of young, physically active females. This limitation reduced the generalizability of the findings.

To date, the effect of CAI on TTB measures in a larger, heterogeneous sample of both males and females has not been investigated. Therefore, the purpose of this study was to compare TTB measures between a mixed-gender group with CAI and a gender-matched control group. We hypothesized that gender would not influence TTB measures of postural control and that a mixed-gender group with CAI would display TTB deficits compared to healthy controls.

## Methods

### Subjects

All subjects were recruited from the student populations at two public universities through research study advertisements. Eighteen males (age: 22.4 ± 5.8 years, height: 179.7 ± 6.8 cm, mass: 84.2 ± 14.9 kg) and fourteen females (age: 20.1 ± 1.9 years, height: 166.1 ± 6.5 cm, mass: 65.6 ± 7.6 kg) with self-reported CAI participated in the study. Inclusion criteria was a history of more than one ankle sprain and self-reported symptoms of disability due to ankle sprains qualified by a score of ≤ 90% on the Foot and Ankle Disability Index (FADI) and ≤ 75% FADI Sport surveys.[18] All CAI subjects had no history of lower extremity injury, including ankle sprain within the past six weeks and no history of lower extremity surgery, balance disorders, neuropathies, diabetes, or other conditions known to affect balance. If a subject reported bilateral ankle instability, the more unstable ankle as reported by the subject was used for analysis. Only the affected or self-reported worse limb of the CAI subjects was tested. CAI subjects reported a mean of 7.8 ± 5.7 ankle sprains with a mean of 10.3 ± 16.4 months since last significant ankle sprain and had a mean FADI score of 86.8 ± 8.2% and a FADI Sport score of 66.5 ± 16.4%.

Eighteen healthy males (age: 23.7 ± 4.6 years, height: 175.6 ± 7.2 cm, mass: 75.4 ± 9.3) and fourteen healthy females (age: 20.8 ± 1.1 years, height: 163.6 ± 6.2 cm, mass: 63.6 ± 10.1 kg) were gender and side matched to the CAI subjects for comparisons. All healthy subjects reported no lower extremity injury in the past year, no history of injury or illness known to affect balance, and no self-reported disability associated with the foot or ankle (100% on the FADI and FADI Sport). Prior to testing, all subjects signed an informed consent form approved by the Institutional Review Board at our institution.

### Procedures

Subjects performed three trials of barefoot single limb stance on each leg with eyes open and closed on a forceplate (Accusway Plus, AMTI, Watertown, MA) for 10 seconds. Subjects were instructed to stand as still as possible during testing with arms folded across their chests, holding the opposite limb at approximately 45° of knee flexion and 30° of hip flexion in accordance with a previously established protocol. [19] All subjects were given one practice trial in each condition to familiarize themselves to the task. If a subject touched down with the opposite limb, made contact with the stance limb, or was unable to maintain standing posture during the 10-second trial, the trial was terminated and repeated. All failed trials were recorded and compared between groups.

### Instrumentation

Postural control was assessed with the Accusway Plus forceplate (AMTI, Watertown, MA). Three dimensional force and moment signals arising from the foot/forceplate interface were filtered using a fourth-order low zero lag, low-pass filter with a cutoff frequency of 5 Hz. COP was calculated from the force and moment signals through Balance Clinic Software (AMTI, Watertown, MA) and sampled at a rate of 50 Hz.[13,15]

### Data Reduction

TTB measures were computed using previously described methods.[15] The mean of three trials for each measure was used for analysis. In order to calculate TTB, each subject's foot was modeled as a rectangle, based on length and width measurements, in order to separate the anteroposterior (AP) and mediolateral (ML) components of COP.[15] TTB measures estimated the time it would take the COP to reach the boundary of the base of support if the COP were to continue on its trajectory at its instantaneous velocity.[15] TTB was processed with the use of a custom software program in MatLab (MathWorks, Inc., Natick, MA). For each COPML data point, the instantaneous position and velocity were used to calculate TTB. The distance between COPML_i _and the previous COPML data point was calculated and divided by the sampling rate (0.02 s) to determine the velocity of COPML_i_. If the COPML_i _was moving medially, the distance from the COPML_i _instantaneous position to the respective (medial) boundary of the foot was determined. By dividing the COPML_i _distance to the boundary by its instantaneous velocity, the theoretical time it would take COPML_i _to reach the medial border of the foot if it continued on the same trajectory without a change in velocity or direction was calculated.[15] If the COP data point was moving laterally, the distance of the COP data point to the lateral border of the foot was determined. TTB in the AP direction was calculated similarly to TTB in the ML direction using the AP borders of the foot rather than the ML. Each TTB series in the ML and AP directions produced a sequence of peaks and valleys. The valleys represented the TTB minima; the lowest values in the TTB series represent the critical times where the sensorimotor system had the least time to make a postural correction in order to maintain single limb stance over the base of support.[15] From the identification of TTB Minima, the absolute minimum TTB, the mean of the TTB minima, and standard deviation of TTB minima were computed separately for the ML and AP directions.

### Statistical Analysis

The means of each dependent measure of the three eyes open and eyes closed trials was used for analysis. Based on analysis of the data using Kolmogorov-Smirnov Z test for normality, all measures were found to be normally distributed, p > 0.05. In order to evaluate whether gender significantly impacted the measures, a series of 2 by 2 analyses of variance were used to determine the effects of group (CAI, control) and gender (male, female) on all TTB variables from the involved limb of the CAI group with eyes open and closed. Because failed trials between groups were not normally distributed, a Mann Whitney U test was used for gender and group comparisons. Alpha level was set a priori at p ≤ 0.05 for all analyses. We opted not to perform any correction for multiple comparisons on the alpha level to protect against type II errors.[20] Instead, effect sizes were generated for each measure by calculating the mean difference between groups and dividing by the standard deviation of the control group. Ninety-five percent confidence intervals (CI) were calculated around each effect size. The strength of effect sizes were classified using Cohen's guidelines.[21] An effect of less than 0.4 was considered small, .41-.7 moderate, and greater than .7 large.

## Results

### Eyes Open Trials

There were no significant group by gender interactions (all p values > 0.16) or main effects for gender (all p values > 0.60) or group (all p values > 0.10) for the TTB measures with eyes open. Group means and standard deviations with corresponding effect sizes are reported in table 1. There was no significant difference in the amount of failed trials during eyes open as all subjects in both groups completed all three trials without failing.

**Table 1:** Group means (± SD) of time-to-boundary (TTB) measures of postural control for eyes open trials.

**Measures**	**CAI (n = 32)**	**Control (n = 32)**	**P-value**	**ES (CI)**
TTBML Absolute Minimum (s)	1.02 ± 0.27	1.10 ± 0.28	0.24	0.29 (0.19 to 0.38)
TTBAP Absolute Minimum (s)	3.42 ± 0.98	3.79 ± 0.99	0.13	0.37 (0.03 to 0.72)
TTBML Mean of Minima (s)	3.77 ± 1.17	4.07 ± 1.54	0.38	0.20 (-0.33 to .73)
TTBAP Mean of Minima (s)	11.61 ± 2.87	12.27 ± 3.75	0.44	0.18 (-1.12 to 1.48)
TTBML SD of TTB Minima (s)	2.87 ± 1.17	3.21 ± 1.88	0.38	0.18 (-0.47 to 0.83)
TTBAP SD of TTB Minima (s)	7.52 ± 2.03	7.70 ± 2.75	0.77	0.07 (-0.89 to 1.02)

CAI = Chronic Ankle Instability, ML = Mediolateral, AP = Anteroposterior, SD = Standard Deviation, ES = Effect Size, CI = 95% Confidence Interval for ES.

### Eyes Closed Trials

There were no significant group by gender interactions (all p values > 0.56) or gender main effects (all p values > 0.25) for any of the TTB measures, but there were significant group main effects for four of the six TTB measures. (see table 2) Significantly lower values were found for the mean of TTBAP minima (p = 0.03), the standard deviation of TTBAP minima (p = 0.03), and the absolute TTBAP minimum (0.03). As well, the CAI group had a significantly lower absolute TTBML minimum (p = 0.03). There were no significant differences in failed trials between genders (p = 0.71). The CAI group also had significantly more failed trials than the control group during the eyes closed condition (control mean: 2.0 ± 0.6, CAI mean: 2.8 ± 0.6, p = 0.04, Effect Size: 1.33 (95% CI: 1.13 to 1.54).

**Table 2:** Group means (± SD) of time-to-boundary (TTB) measures of postural control for eyes closed trials.

**Measures**	**CAI (n = 32)**	**Control (n = 32)**	**P-value**	**ES (CI)**
TTBML Absolute Minimum (s)	0.48 ± 0.10	0.53 ± 0.10	0.03	0.50 (0.47 to 0.53)
TTBAP Absolute Minimum (s)	1.36 ± 0.40	1.61 ± 0.47	0.03	0.53 (0.37 to 0.69)
TTBML Mean of Minima (s)	1.82 ± 0.51	2.04 ± 0.58	0.11	0.38 (0.18 to 0.58)
TTBAP Mean of Minima (s)	4.71 ± 1.14	5.51 ± 1.63	0.03	0.49 (-0.07 to 1.06)
TTBML SD of TTB Minima (s)	1.76 ± 0.86	1.84 ± 0.63	0.66	0.13 (-0.09 to .35)
TTBAP SD of TTB Minima (s)	2.97 ± 0.84	3.53 ± 1.21	0.03	0.46 (0.04 to 0.88)

CAI = Chronic Ankle Instability, ML = Mediolateral, AP = Anteroposterior, SD = Standard Deviation, ES = Effect Size, CI = 95% Confidence Interval for ES.

## Discussion

The primary finding of this study was the identification of significant TTB deficits during the eyes closed balance trials in a mixed-gender CAI group in comparison to a mixed-gender control group. When vision was removed, the mean of TTB minima and the standard deviation of TTB minima in the AP direction significantly decreased in the CAI group. This indicated that the CAI group moved closer to the spatiotemporal boundaries of stability in a more predictable manner compared to the controls. Overall, the CAI group had significantly less time to make a postural correction in the AP direction than the control group. These results are somewhat consistent with previous findings of TTB postural impairments in females with CAI,[13] however, in the current study TTB differences were not identified in the eyes open trials whereas they were previously. As we hypothesized, gender did not have a significant influence on TTB measures.

Previously, Hertel and Olmsted-Kramer[13] identified significant TTB deficits in females in single limb stance with eyes open. Five out of the six TTB measures were significantly decreased in the CAI group and only one of the traditional measures (COP ML Velocity) was significantly different. The CAI group in the present study reported a mean FADI score of 86.8 ± 8.2% and a FADI Sport score of 66.5 ± 16.4%. Because there was no standardized report of disability in the previous study,[13] there was no way of knowing whether or not these two CAI groups reported similar disability. Possibly, in our study, the CAI subjects may not have been as significantly impaired as in the previous study. The means and standard deviations of all TTB measures for the eyes open trials in our study were twice as large as the previous study. Because there were no significant differences between genders in our study, it may be that the severity of self-reported disability significantly influences these measures. Effect size calculations for the TTB measures of eyes open trials were small in this study. It was apparent that an impairment of postural control due to CAI was not detected in the presence of visual input.

When vision was removed, moderate effect sizes were found for the TTB absolute minimum (ML effect size = 0.50, AP effect size = 0.53) and small to moderate for the means of TTB minima (ML effect size = 0.38, AP effect size = 0.50) in the ML and AP directions. The effect sizes for the standard deviation of TTB minima were small to moderate in this study (ML effect size = 0.13, AP effect size = 0.46) compared to large effect sizes in the previous study.[13] Although our statistical analysis resulted in significant decreases in the absolute TTB minimum in the ML and AP directions, the small to moderate effects of CAI on the mean of TTB minima in both the ML and AP directions are noteworthy. Based on the 95% confidence intervals, statistically significant deficits may have been detected with a larger sample size at this level of self-reported disability. An interesting observation was the 95% confidence interval for the effect size of the mean of TTBAP minima with eyes closed. The confidence interval (-0.07 to 1.06) crossed zero, which indicated uncertainty as to whether a true difference was actually detected. Upon further inspection, the width of the confidence interval was influenced by the standard deviation in the control group (1.67) compared to the CAI group (1.14). This finding appears to be in agreement with diminished standard deviation of TTBAP minima in the CAI group compared to the control. The confidence interval around the effect of that significant difference did not cross zero (0.04 to 0.88).

We did not find significant differences in the mean or standard deviation of the TTBML minima with eyes closed. A potential limitation may be that the boundaries used in the calculation of TTBML underestimate the actual time it would take the COP to reach the medial or lateral boundaries of the foot. The widest part of the foot, near the metatarsal heads was used to construct the ML boundary. The COP was generally situated posterior to the metatarsal heads in a narrower portion of the foot. There may be a deficit in the ML direction; however the current TTB model may have lacked the sensitivity to detect it.

This is the first study to demonstrate significant TTB deficits with the concomitant removal of vision and CAI. In both groups, when vision was removed, TTB measures significantly decreased in both directions compared to eyes open (p < 0.001). This indicates that the removal of vision significantly constrained the sensorimotor system's ability to maintain postural control over a narrow base of support. Only with the removal of vision did the TTB differences between groups manifest themselves. When vision was removed, the CAI group had a greater number of failed trials compared to the control group. The large effect size with narrow confidence intervals is indicative of a true difference in the ability to maintain single limb stance without visual input in those with CAI compared to the controls.

We speculate that the TTB deficits detected in the CAI group without vision may be related to unique organismic constraints placed upon the sensorimotor system arising from altered somatosensory input. It may be that damage to the articular and ligamentous receptors of the ankle introduce a constraint to the sensorimotor system limiting the ability to find effective postural control strategies. In TTB measures, the standard deviation of TTB minima indicates the level of constraints on the sensorimotor system.[22] When vision was removed in both groups, the standard deviation of TTB minima in the ML and AP directions significantly decreased compared to when visual feedback was available. Similarly, CAI may also significantly constrain this ability to find effective movement strategies. This may be reflective of the greater number of failed trials in the CAI group. From this perspective, the altered TTB behavior observed in the CAI group may arise from the unique interactions between an impaired sensorimotor system and the task of maintaining single limb stance in the absence of visual input.

## Conclusion

Postural control deficits as assessed with TTB measures were present in the CAI group compared to the control group when balance testing was performed with eyes closed, but not eyes open. CAI may place substantial constraints on the sensorimotor system during prolonged single limb stance, resulting in a reorganization of postural control strategies. These deficits may be indicative of a diminished ability to respond effectively to changes in postural control demands in those with CAI.

## Pre-publication history

The pre-publication history for this paper can be accessed here:



## References

[B1] Beynnon BD, Renstrom PA, Alosa DM, Baumhauer JF, Vacek PM (2001). Ankle ligament injury risk factors: a prospective study of college athletes. J Orthop Res.

[B2] Beynnon BD, Murphy DF, Alosa DM (2002). Predictive Factors for Lateral Ankle Sprains: A Literature Review. J Athl Train.

[B3] Bahr R, Lian O, Bahr IA (1997). A twofold reduction in the incidence of acute ankle sprains in volleyball after the introduction of an injury prevention program: a prospective cohort study. Scand J Med Sci Sports.

[B4] Bahr R, Bahr IA (1997). Incidence of acute volleyball injuries: a prospective cohort study of injury mechanisms and risk factors. Scand J Med Sci Sports.

[B5] Hertel J (2002). Functional Anatomy, Pathomechanics, and Pathophysiology of Lateral Ankle Instability. J Athl Train.

[B6] Baier M, Hopf T (1998). Ankle orthoses effect on single-limb standing balance in athletes with functional ankle instability. Arch Phys Med Rehabil.

[B7] Isakov E, Mizrahi J (1997). Is balance impaired by recurrent sprained ankle?. Br J Sports Med.

[B8] Perrin PP, Bene MC, Perrin CA, Durupt D (1997). Ankle trauma significantly impairs posture control – a study in basketball players and controls. Int J Sports Med.

[B9] Rozzi SL, Lephart SM, Sterner R, Kuligowski L (1999). Balance training for persons with functionally unstable ankles. J Orthop Sports Phys Ther.

[B10] McKeon PO, Hertel J (2008). Systematic review of postural control and lateral ankle instability, part 1: Can deficits be detected with instrumented testing?. J Athletic Training.

[B11] Bernier J, Perrin D, Rijke A (1997). Effect of unilateral functional instability of the ankle on postural sway and inversion and eversion strength. J Athletic Training.

[B12] Cornwall MW, Murrell P (1991). Postural sway following inversion sprain of the ankle. J Am Podiatr Med Assoc.

[B13] Hertel J, Olmsted-Kramer LC (2007). Deficits in time-to-boundary measures of postural control with chronic ankle instability. Gait Posture.

[B14] van Wegen EE, van Emmerik RE, Riccio GE (2002). Postural orientation: age-related changes in variability and time-to-boundary. Hum Mov Sci.

[B15] Hertel J, Olmsted-Kramer L, Challis J (2006). Time-to-boundary measures of postural control during quiet single leg standing. J Appl Biomech.

[B16] van Emmerik R, van Wegen E (2002). On the functional aspects of variability in postural control. Exercise and Sport Sciences Reviews.

[B17] van Wegen EE, van Emmerik RE, Wagenaar RC, Ellis T (2001). Stability boundaries and lateral postural control in parkinson's disease. Motor Control.

[B18] Hale SA, Hertel J (2005). Reliability and Sensitivity of the Foot and Ankle Disability Index in Subjects With Chronic Ankle Instability. J Athl Train.

[B19] Hertel J, Buckley WE, Denegar CR (2001). Serial Testing of Postural Control After Acute Lateral Ankle Sprain. J Athl Train.

[B20] Perneger TV (1998). What's wrong with Bonferroni adjustments. Bmj.

[B21] Cohen J (1988). Statistical Power Analysis for the Behavioral Sciences.

[B22] Haibach PS, Slobounov SM, Slobounova ES, Newell KM (2007). Virtual time-to-contact of postural stability boundaries as a function of support surface compliance. Exp Brain Res.

